# Pollen-Pistil Interaction

**DOI:** 10.3390/ijms24043707

**Published:** 2023-02-13

**Authors:** Giampiero Cai, Iris Aloisi, Stefano Del Duca

**Affiliations:** 1 Department of Life Sciences, University of Siena, 53100 Siena, Italy; 2 Department of Biological, Geological and Environmental Sciences, University of Bologna, 40126 Bologna, Italy; 3 Interdepartmental Centre for Agri-Food Industrial Research, University of Bologna, 40126 Bologna, Italy

The aim of this Special Issue is to highlight the molecular dialogue between the pollen tube and the pistil. This is achieved with original articles and reviews which show how this dialogue is controlled at the genomic and molecular levels. During the angiosperm’s double fertilization, the pollen tube must enter female tissues, bypass numerous physical barriers to reach the micropyle, and release gametes to complete the fertilization process with the final fusion between male and female gametes. There are molecular signals produced by the pistil that are intercepted by the receptors located primarily at the tip of the tube, which generate effects that modulate its growth activity; in turn, the pollen tube releases molecules that determine effects on the pistil cells. Thus, a complex dialogue develops between the female and male counterparts, whose language is made up of an expansive molecular alphabet that includes proteins, glycoproteins, arabinogalactan-proteins, lipid-binding proteins, nanovesicles, ions, amino acids, sugars, hormones, reactive oxygen species (ROS), and modulators of gene expression.

The dialogue between the pistil and the pollen tube is more complex in self-incompatible species, which can reject the self-incompatible pollen to promote genetic diversity. Self-incompatibility (SI) is complex and characterized by specific proteins that must interface with the signal transduction system, cytoskeleton, and all the molecular factors mentioned above [1].

Additionally, the impact that climate change can have on the molecular dialogue between pistil and pollen tube should not be overlooked or underestimated. Environmental stresses such as changes in temperature, water availability, salinity, and UV radiation can affect one or more steps in the pistil-pollen grain and/or pollen tube cross-talk, leading to missed or undesired plant fertilization and reproduction [2]. It is also important to note that some proteins released from pollen grains and pollen tubes are allergens that could be present at higher levels and with increased sensitizing capacity, under conditions of climate change, thereby increasing health problems.

The ability of the pollen tube to overcome various physical barriers through its dialogue with the pistil has been addressed by Robichaux and Wallace [3]. In this interesting article, the authors pointed out that, during its journey, the pollen tube overcomes substantive physical barriers, including the penetration of the stigmatic papillae, style, transmission tract, and synergid cells, as well as the fusion of the sperm with the ovule. Furthermore, the pollen tube is able to maintain its structural integrity in these compact environments while responding to positional orientation cues that guide the pollen tube to its destination. Communication between the pollen and pistil tissues is essential to mediate these events and is achieved through the expression of certain genes and various specific molecules. This is the example of γ-aminobutyric acid (GABA), whose gradient along the pistil can serve as a signal for positional orientation, and arabinogalactan-proteins rich in hydroxyproline (AGP), which are particularly abundant in the cells of the female tissue, that promote germination and the elongation of the pollen tube.

Interspecific reproductive barriers (IRBs) are present in the tomato, *Solanum lycopersicon*. In this genus, authors Muñoz-Sanz et. al [4] observed that the incompatible species *S. pennelli* blocks *S. lycopersicon* pollen, which only reaches the upper third of the pistil. Using a CRISPR/Cas9 approach, eight genes responsible for this reproductive barrier were identified; one of these genes encodes for a small cysteine-rich protein. The lack of expression of this gene allows the pollen to penetrate more deeply, reaching the lower part (bottom third) of the pistil. This suggests that this gene is involved in controlling this barrier without involving RNAase, as occurs in many systems characterized by gametophytic SI.

The control of tube growth by protein factors was addressed by Mei and collaborators [5] who observed in *Arabidopsis thaliana* how the temporal control of pollen germination before the pollen grain reaches the stigma is critical for successful fertilization in angiosperms. However, the mechanisms underlying this process remain poorly understood. Among the factors involved are MAPKinases, which play a role in the control of the germination process. Increased expression of these genes has negative effects on reproduction, being responsible for callose accumulation, increase in jasmonic acid (JA) and early pollen germination, leading to a significant reduction in seed formation.

Other authors verified the involvement of aspartic acid-rich proteins (ASP-RICH) during SI response since these proteins are differentially expressed in compatible and self-incompatible crosses (SI) in clementines [6]. To this end, the ASP-RICH proteins were expressed in tobacco plants, where the expression of these proteins modulated the Ca^2+^ content and consequently affected the organization of the cytoskeleton and the deposition of cell wall components, having consequences on the growth of pollen tubes. Although the expression of ASP-RICH proteins did not exert a marked effect on the growth rate of pollen tubes, effects at the level of growth pattern suggest that ASP-RICH expression may have a regulatory action on the growth mechanism of pollen tubes.

In the genus Turnera, the pollen-pistil interaction is controlled by a single S locus containing three genes, one of which is *TsBAHD,* which encodes a protein expressed in the pistils that has conserved motifs found in BAHD acyltransferases. Mutant and wild-type BAHD alleles were expressed in *Arabidopsis thaliana* to test whether brassinosteroids (BR) directly act in SI. BRs were added to *Arabidopsis thaliana* pollen cultures in vitro and a small specific stimulatory effect on pollen tube growth was detected with 5 µM brassinolide, suggesting that BAHD acts pleiotropically to mediate pistil length and physiological mating type through the inactivation of BR. Thus, in the SI context, BR act by differentially regulating gene expression in pistils, rather than directly on pollen [7].

One interesting article analyzes the latest findings on SI in orchids at the morphological, physiological, and molecular levels. In Orchidaceae, SI is mostly found in the subfamily Epidendroideae, and the SI phenotypes are diverse, even within the same genus. Hormones, i.e., auxin and ethylene, and new male and female determinants could be involved in the SI response [8].

In another experimental model represented by kenaf (*Hibiscus cannabinus* L.), the genes controlling cytoplasmic male sterility (CMS) were studied [9]. CMS, the inability of plants to produce functional anthers, pollen, or male gametes, is under extranuclear genetic control (by mitochondrial or plastid genomes). CMS is extensively exploited in the production of hybrid seeds that are more uniform, and have considerably higher yields than open-pollinated plants, as well as other desirable traits, such as disease, drought, and weather resistance. In kenaf, it has been observed that male sterility depends on gene expression, hormonal biosynthesis (Indole-3-acetic acid, abscisic acid (ABA)), and the synthesis of molecules with a high-energy content (photosynthesis products and ATP). In the infertile versus control male line, DNA methylome analysis revealed 650 differentially methylated genes with 313 up and 337 down-methylated genes, whereas transcriptome analysis revealed 1788 differentially expressed genes with 558 up and 1230 downregulated genes. Differentially expressed genes important for CMS in kenaf are involved in carbon metabolism, plant hormone signaling, the tricarboxylic acid (TCA) cycle, and the MAPK signaling pathway.

In rice, RNA sequencing technology was used to study transcriptome changes during double fertilization. The following involved genes were identified: 1669 genes, related to the guided growth of pollen tubes, 332 genes involved in male-female gamete recognition and fusion, and 430 genes related to the zygote formation and to the first nuclear division of free endosperm. Among 1669 pollen tube-related genes, seven arabinogalactan proteins (AGP), one cysteine-rich peptide (CRP), and fifteen receptor-like kinases (RLK) were found to be specifically expressed in the anther, while two AGP, seven CRP, and five RLK were found in the pistil, showing an evident unequal distribution of these factors that could play different roles in the anther and pistil during fertilization [10].

To characterize the transcriptome of mature pollen in maize and identify its specific genes, RNA-seq-data during maize tissue development reported in an atlas as publicly available was used for meta-analyses. This allowed the identification of pollen and anther specific genes. Data were also evaluated with Gene Ontology (GO) and Kyoto Encyclopedia of Genes and Genomes pathway (KEGG), allowing the identification of 1215 pollen-specific mature genes (MPS) and 1009 anther-specific mature genes (MAS); of these 623 were present in both datasets and are the mature anther and pollen-specific genes (MAPS), i.e., late-stage pollen-specific genes of the maize genome. 71% of these genes encode for pollen-allergenic proteins. Interestingly, analysis of the promoters of the MAPS genes identified elements that respond to hormones such as JA and ABA, suggesting the involvement of these hormones in pollen-specific MAPS expression. This study describes a rapid method for discovering pollen-specific promoters from publicly available data while providing farmers and the corn industry with a number of MAPS promoters that can be targeted for use in Seed Production Technology to produce hybrid varieties by increasing crop yield [11].

The effects of environmental stresses on pollen-pistil interactions were presented in a review article and in an original research article on cotton. In the review article, the authors described the main effects that environmental stresses can cause on pollen, such as thermal stress induced by high and low temperatures, heavy metals, UV-B radiation, osmotic stress, and nutrient deficiency [12]. The most relevant evidence underlying the morphological, cytoskeletal, metabolic, and signaling alterations involved in the perception and response to stress were illustrated, focusing on the final stage of pollen life, i.e., from hydration, pollen tube growth, and transport of sperm cells, these being the phases most sensitive to environmental climatic changes. In the response to stress and in adaptation, a protective and mitigating action of the induced stress is carried out by polyamines, known molecules involved in the growth and development of plant cells [13] which may exert a protective action against environmental stress by promoting pollen development and pollen tube growth. Moreover, polyamines are involved in SI response [14,15].

In cotton, high-temperature stress inhibits the opening of the anthers resulting in sterility and consequently causing a low yield of the crop. The authors developed a transcriptomic profiling analysis system that quickly identified the genes involved in the response to high-temperature stress. Some genes have been identified as being involved in the stress response in addition to being regulated by the hormone methyljasmonate (MeJA); these are about ten genes that are responsible for the plant’s response to high temperatures stress. The study could serve as an example of an experimental approach to quickly identify the genes involved in a given process [16].

Finally, it is clear that the dialogue between the pollen and the pistil is crucial for many biological events and applications, and it is also clear that a deeper knowledge of this interaction is required for the understanding and improvement of plant reproduction under standard climate conditions and even more so under future climate changes. This Special Issue provides an insight into how both basic and applied research are contributing to the understanding of the molecular dialogue between pollen and pistil, which is crucial for many biological events and applications.

## Data Availability

The data presented in this study are available on request from the corresponding author.
